# QTL Mapping in Three Rice Populations Uncovers Major Genomic Regions Associated with African Rice Gall Midge Resistance

**DOI:** 10.1371/journal.pone.0160749

**Published:** 2016-08-10

**Authors:** Nasser Yao, Cheng-Ruei Lee, Kassa Semagn, Mounirou Sow, Francis Nwilene, Olufisayo Kolade, Roland Bocco, Olumoye Oyetunji, Thomas Mitchell-Olds, Marie-Noëlle Ndjiondjop

**Affiliations:** 1 Biosciences eastern and central Africa (BecA), Nairobi, Kenya; 2 Institute of Ecology and Evolutionary Biology, National Taiwan University, Taipei, Taiwan, ROC; 3 Institute of Plant Biology, National Taiwan University, Taipei, Taiwan, ROC; 4 Department of Agriculture, Forestry and Nutrition Science, University of Alberta, 4–10 Agriculture/Forestry Centre, Edmonton, Canada; 5 AfricaRice, 01 BP 2031, Cotonou, Benin; 6 AfricaRice, PMB 5320, Ibadan, Oyo State, Nigeria; 7 Institute for Genome Sciences and Policy, Department of Biology, Duke University, Durham, North Carolina, United States of America; Mahatma Phule Krishi Vidyapeeth College of Agriculture, INDIA

## Abstract

African rice gall midge (AfRGM) is one of the most destructive pests of irrigated and lowland African ecologies. This study aimed to identify the quantitative trait loci (QTL) associated with AfRGM pest incidence and resistance in three independent bi-parental rice populations (ITA306xBW348-1, ITA306xTOG7106 and ITA306xTOS14519), and to conduct meta QTL (mQTL) analysis to explore whether any genomic regions are conserved across different genetic backgrounds. Composite interval mapping (CIM) conducted on the three populations independently uncovered a total of 28 QTLs associated with pest incidence (12) and pest severity (16). The number of QTLs per population associated with AfRGM resistance varied from three in the ITA306xBW348-1 population to eight in the ITA306xTOG7106 population. Each QTL individually explained 1.3 to 34.1% of the phenotypic variance. The major genomic region for AfRGM resistance had a LOD score and *R*^2^ of 60.0 and 34.1% respectively, and mapped at 111 cM on chromosome 4 (*qAfrGM4*) in the ITA306xTOS14519 population. The meta-analysis reduced the number of QTLs from 28 to 17 mQTLs, each explaining 1.3 to 24.5% of phenotypic variance, and narrowed the confidence intervals by 2.2 cM. There was only one minor effect mQTL on chromosome 1 that was common in the TOS14519 and TOG7106 genetic backgrounds; all other mQTLs were background specific. We are currently fine-mapping and validating the major effect genomic region on chromosome 4 (*qAfRGM4*). This is the first report in mapping the genomic regions associated with the AfRGM resistance, and will be highly useful for rice breeders.

## Introduction

Rice (*Oryza* spp.) is a staple food for millions of people in Africa. According to FAO 2014 data (http://faostat3.fao.org), the overall paddy rice yield in sub Saharan Africa has increased from 2.2 t ha^–1^ in 2000 to 2.7 t ha^–1^ in 2013, which is very low compared with the 2013 average yield reported in Asia (4.6 t ha^–1^), South America (5.2 t ha^–1^) and North America (8.6 t ha^–1^). Several factors–including high incidence of insect pests, diseases, drought, poor soil fertility, limited irrigation, and farmers’ inability to afford fertilizers–have contributed to low productivity in sub-Saharan Africa. African rice gall midge (AfRGM), *Orseolia oryzivora* Harris and Gagné, is one of the most destructive pests of irrigated and lowland ecologies across 19 African countries [1]. It is indigenous to Africa and morphologically distinct from Asian rice gall midge (AsRGM), *Orseolia oryzae* Wood-Mason. Crop damage is caused by the larvae [2], which infest rice tillers at the vegetative growth stage and destroy the growing primordia. Such larval infestation results in the formation of galls in the plants and prevents tillers from developing more leaves or panicles.

AfRGM is an endemic pest to Africa and it was first reported in Sudan [3]. Currently, the pest is spreading throughout Africa and found in 12 West African, two Central African and five East and Southern African countries [4]. The insect pest causes 20 to 100% yield losses in the worst-affected areas [1, 2, 5–9], with the extent of damage depending on several factors, including climatic conditions (high rainfall, excessive cloud cover and high humidity), ecosystem (rainfed lowland, hydromorphic, upland and mangrove ecologies), planting season, type of germplasm (landraces vs. improved varieties), planting method (direct seeding vs. transplanting), plant population density, and cultural practices. One percent of infested tillers can cause a 2% yield loss [10], and in Nigeria, a 1% increase of infestation resulted in a 2.9% yield loss [1, 9]. In certain regions, severe attacks lead to total loss of the harvest [6].

AfRGM can be controlled using a wide range of methods, including biological, chemical and cultural control strategies, but host-plant resistance is the most effective, durable and farmer-friendly control measure against this pest [11, 12]. Many rice varieties currently available to farmers are highly susceptible to AfRGM. Improving varietal resistance appears to be one of the most promising options for managing the pest, especially in Asia where resistant varieties have been used with considerable success against AsRGM. Therefore, since the early 1980s, rice varieties have been screened for resistance to AfRGM in Nigeria by the National Cereals Research Institute (NCRI), in collaboration with the Africa Rice Centre (AfricaRice), International Rice Research Institute (IRRI) and the International Institute of Tropical Agriculture (IITA). Despite intensive screening, no *O*. *sativa* lines have been found with very strong resistance under high AfRGM pressure. However, a number of *O*. *glaberrima* varieties with relatively better resistance to AfRGM have been identified, which includes TOG7106 [11]. Most of these traditional varieties are low yielding and unsuitable for large-scale cultivation.

The identification of genes or quantitative trait loci (QTL) with consistently large phenotypic effects across genetic backgrounds and environments is one of the prerequisites for rice improvement for AfRGM resistance using marker assisted selection (MAS). The identification and utilization of genes or QTLs conferring resistance to AsRGM has been a major objective of rice breeding in Asia. Thus far, at least eleven genes associated with AsRGM resistance have been identified and characterized [13, 14]; the flanking molecular markers associated with some of these genes have been used in MAS programs for developing AsRGM resistant varieties [15, 16]. However, these genes have not been evaluated for their response to the AfRGM, nor have other similar studies identified genes or QTLs associated with AfRGM resistance. This forms the basis of the present study. Phenotypic results from multi-location screening of a wide range of *O*. *glaberrima* and *O*. *sativa* germplasm for AfRGM response have helped rice breeders to identify several varieties with a range of responses to AfRGM [2, 5, 11, 12, 17–19]. Based on quantitative comparisons of relative damage levels, we have developed mapping populations using TOG7106, TOS14519 and BW348-1, which are considered to be resistant, moderately resistant and tolerant to AfRGM, respectively [11, 17, 19, 20]. Neither the genes or QTLs associated with resistance to AfRGM, nor their mode of action and phenotypic effects on these varieties, had yet been discovered.

The traditional method of exploiting molecular markers in MAS involves finding a subset of markers that are significantly associated with one or more of the QTLs that regulate the expression of complex traits in bi-parental populations. In cases where QTL mapping studies are carried out across multiple populations, researchers usually attempt to determine if the QTLs identified in each are the same either by comparing the chromosomal position of a common subset of markers across different studies, and/or indirectly, by comparing each mapping population to a reference map [21]. Co-localized QTLs may not be identical, however, especially when they are associated with large confidence intervals. Meta-QTL (mQTL) analysis [22] is a better method for combining data from independent studies to detect consensus QTLs and to shrink QTL confidence intervals. Meta-analyses have been reported in rice [23, 24] and several other crops, such as maize, wheat, rapeseed, potato, cotton, soybean, barley, cocoa and apricot [25, 26]. In most QTL meta-analyses published so far [23, 24, 26–28], authors have compiled published linkage maps and QTL results from independent studies using different phenotyping protocols, constructed consensus linkage maps using a subset of markers common to the different studies, and projected mQTLs positions and their confidence intervals onto the consensus map. Limitations of these studies include the use of different phenotyping protocols, different QTL mapping methods and parameters; and use of too few common markers, lowering confidence in the mQTL and the delimited intervals.

The use of single nucleotide polymorphism (SNP) markers has emerged as a powerful tool for many genetic applications due to low assay costs, high genomic abundance, locus-specificity, co-dominant inheritance, potential for high throughput analysis and relatively low genotyping error rates [29, 30]. SNP data can be obtained using one of the numerous uniplex or multiplex SNP genotyping platforms that combine a variety of chemistries, detection methods and reaction formats [31, 32]. Kompetitive Allele Specific PCR (KASP) is a uniplex SNP genotyping platform, which offers cost-effective and scalable flexibility in applications requiring small to moderate numbers of markers, with genotyping error rate < 1.6% [33]. The objectives of the present study were (a) to identify SNPs and QTLs associated with AfRGM resistance in three bi-parental rice populations genotyped with a common SNP platform using KASP and phenotyped with a common protocol; and (b) to combine the results of all QTLs detected across the three populations through a meta-QTL analysis, and explore whether there are any genomic regions conserved across different genetic backgrounds.

## Material and Methods

### Development of mapping population

The present study was based on three mapping populations derived from a cross between ITA306 (female parent) and TOG7106, TOS14519 and BW348-1 (male parents). The general crossing scheme is shown in S1 Fig. ITA306 is a popular, high-yielding *Oryza sativa* L. subsp. *indica* variety, released in Nigeria but highly susceptible to AfRGM. TOG7106, TOS14519 and BW348-1 are *O*. *glaberrima* Steud., *O*. *sativa* subsp. *japonica* and *O*. *sativa* subsp. *indica* varieties, respectively. TOG7106 is rated as highly resistant or resistant, while TOS14519 and BW348-1 were considered as moderately resistant and tolerant to AfRGM, respectively [11, 17, 34]. F_1_ seeds were dehulled manually and then pre-germinated in petri dishes. Each seedling was then transplanted into a separate 12-liter plastic bucket filled with paddy soil. For quality control analysis, leaf samples were collected from each F_1_ plant, DNA extracted and genotyped with 100 SNPs that were polymorphic between the two parents using KASP assay. F_1_ plants that passed the parent-offspring tests with ≥ 90% matches with the expected parental alleles were advanced to the next generation; other plants were discarded. For intra-species cross ITA306xTOS14519, F_1_ plants were self-fertilized to generate F_2_ plants, and 615 F_2_ plants were self-fertilized again to generate 615 F_2:3_ families, one for each F_2_ plant. The intra-species cross ITA306xBW348-1 had the same design and results in 150 F_2:3_ families. For the inter-specific cross involving ITA306xTOG7106, we used a backcross design due to F_1_ pollen sterility. F_1_ plants were used as females and back-crossed to the susceptible ITA306 parent, generating 87 BC_1_F_1_ plants. The BC_1_F_1_ plants were self-fertilized to generate 87 BC_1_F_2_ families. We pooled DNA for genotyping and measured phenotypes together from multiple plants of the same family, representing the genotypes and phenotypic means of their F_2_ (for the two intra-specific crosses) or BC_1_F_2_ (for the inter-specific cross) families.

### Phenotyping

The AfRGM colony was reared in a screen-house at the International Institute of Tropical Agriculture (IITA), Ibadan, Nigeria. It was reared on the susceptible ITA306 plants, by timing the plant age for infestation with emergence of adult midges from the culture plants. The mapping populations were transplanted into the paddy screen-house in blocks of 10 test entries with two checks (resistant–TOS14519, and susceptible–ITA306). Seeds were sown on a nursery bed and transplanted into a paddy screen house 14 days after sowing. Transplanting was done in 2 m rows, with an equal spacing of 20 cm between rows and plants, using an augmented experimental design. Each test entry (family) consisted of 20 hills or plants. The two checks were sown after every 10 testing entries to (i) assess the mapping population relative to a nearby resistant or susceptible check, and (ii) to verify an even distribution of the gall midge population throughout the trial. An infestation band comprising of five rows of the susceptible ITA306 parent, which was also used as susceptible check, flanked the experimental plot. An equivalent of 40 kg ha^–1^ each of N, P_2_O_5_ and K_2_O was applied at transplanting using NPK 15–15–15 (% a.i.). An additional 40 kg urea ha^–1^ was applied 21 days after transplanting. The plots were hand-weeded at 21 and 40 days after transplanting. The pest evaluation experiments were repeated twice, each in three replications, the phenotypic traits studied was implemented as described by [20].

### Genotyping

Genomic DNA was extracted, using the cetyltrimethyl ammonium bromide (CTAB) method [35] with minor modifications, from 2–3 week old fresh leaves collected from a bulk of 10 plants from each F_2:3_ and BC_1_F_2_ family. The quality of the isolated DNA was checked by running aliquots of DNA samples on a 1% agarose gel that contained 0.5 μg/mL GelRed. DNA concentration was measured using a SmartSpec Plus spectrophotometer (Bio-Rad, USA) as described in the user’s manual, then normalized and shipped to the LGC Genomics (http://www.lgcgenomics.com) genotyping laboratory in UK. DNA samples from ITA306xTOS14519, ITA306xTOG7106, and ITA306xBW348-1 mapping populations were genotyped with 164, 221 and 417 polymorphic SNPs using KASP assay, respectively.

### Statistical analyses

For each trait, analysis of variance (ANOVA) was performed using the general linear model (GLM) implemented in Minitab v.14 (Minitab Inc., State College, PA, USA). Tests for normality and the frequency distribution of means were also done using MiniTab v.14. Broad sense heritability was computed using lme4 package implemented in the Multi Environment Trait Analysis with R for Windows (MetaR) v.4.1. Genotypes (entries) were considered as fixed, while replications and trials (experiments) were considered as random. Chi-square analyses on the genotypic data and linkage mapping were performed using JoinMap version 4.0 [36] as described [37]. For the same chromosome across the three populations, a consensus linkage map of all SNPs was constructed from the population specific maps using BioMercator v.40 [38]. Composite interval mapping (CIM) was performed on the mean values of each entry using PLABQTL version 1.2 [39] with the following parameters: a minimum LOD score of 2.5, automatic cofactor selection, walking speed of 1 cM, a model to determine additive effects at individual QTL and additive x additive epistatic interactions, and F-to-Enter value of ten [40]. QTL names were designated following the rice QTL nomenclature rules [41]. Linkage and QTL maps were drawn using MapChart v2.1 [42]. In this study, QTL that explained <10% and ≥10% of the total phenotypic variation (R^2^) were classified into minor and major effect QTL, respectively.

### Map projection and QTL meta-analyses

**Fig 1:**
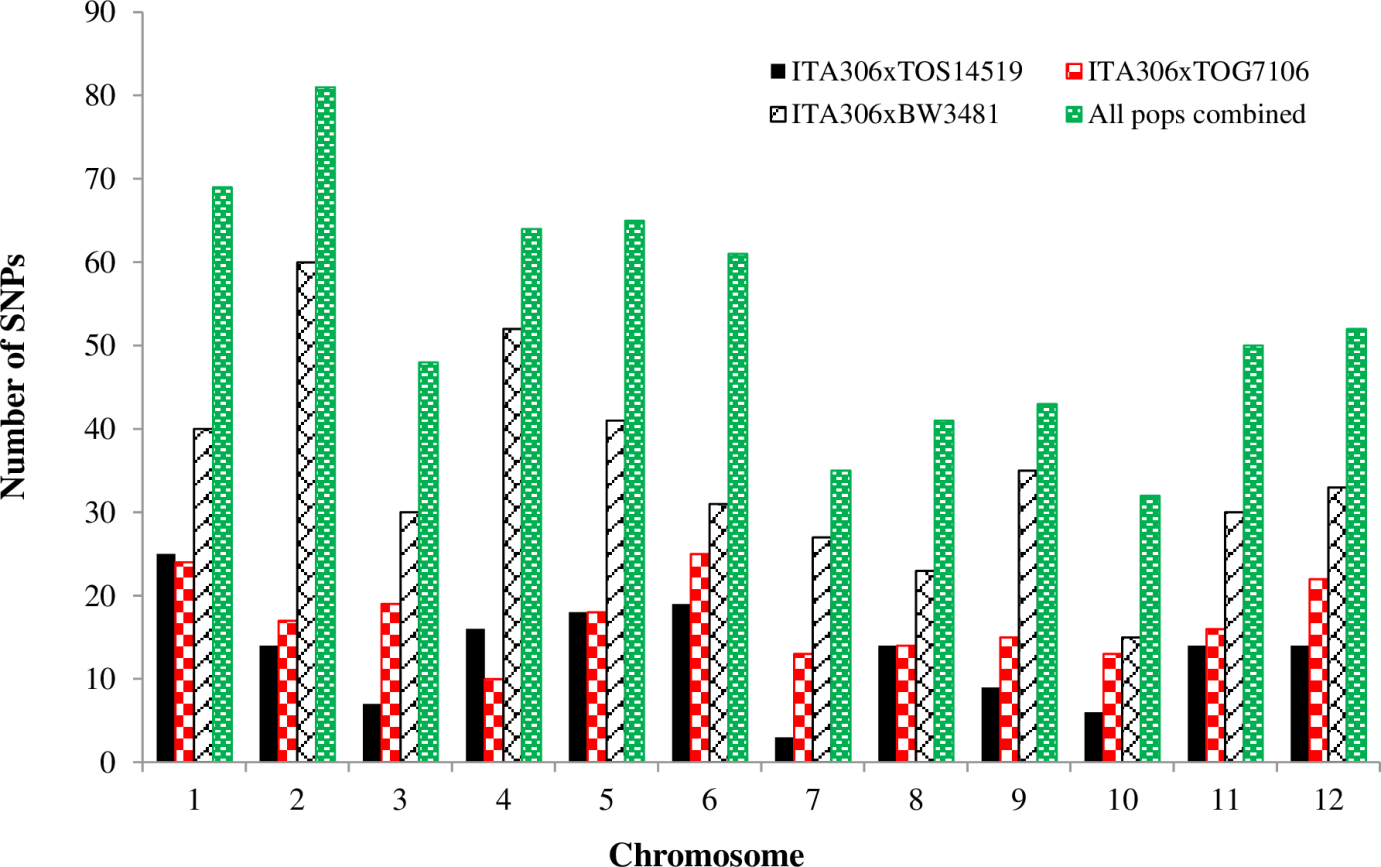
Summary of the number of SNPs used for QTL mapping for each of the three populations and combined across all populations.

All QTL identified in the individual populations using CIM were projected on the consensus linkage maps of the 641 SNPs (Fig 1, S1 Table) using the chromosomal position, LOD score, confidence interval and proportion of phenotypic variance (*R*^2^) explained by each QTL (as summarized in Table 1). For each chromosome, meta-analysis was used to estimate the numbers, positions and 95% confidence interval of the mQTL using BioMercator v.4 (https://urgi.versailles.inra.fr/Tools/BioMercator-V4) [43], as described by Semagn et al. [37]. The first stage of QTL meta-analysis determines the model that best corresponds to the real number of QTL, based on the following criteria: Akaike information criterion (AIC), AICc, AIC3, Bayesian information criterion (BIC) and average weight of evidence (AWE). The best QTL model was selected when values of the model selection criteria were the lowest at least in three of the five models (S2 Table). The best model was then used in the second step of the QTL meta-analysis. QTL with probability of membership in a given mQTL > 60% were assigned to the same mQTL.

**Table 1:** Summary of the population-specific QTLs associated with pest incidence (PI) and pest severity (PS) in three mapping populations.

QTL	Population	Trait*	Chrom	Position (cM)	Confidence interval (cM)	Left flanking marker	Right flanking marker	LOD	R^2^ (%)	Additive effect	Dominant effect	Difference**
*qAfRGM1*.*1*	ITA306xBW348-1	PS	1	17	14–18	K_id1002308	id1002863	3.0	6.8	0.5	6.8	1.5
*qAfRGM1*.*2*	ITA306xTOS14519	PS	1	134	124–138	K_id1014452	K_id1020631	8.4	4.3	-4.3	-2.0	-11.5
*qPI1*.*1*	ITA306xTOS14519	PI	1	138	136–140	K_id1020828	K_id1022408	2.8	1.6	-5.6	-9.7	-29.8
*qAfRGM1*.*3*	ITA306xTOG7106	PS	1	147	142–153	K_id1020667	K_id1024503	3.0	2.8	-12.5	-33.8	-25.7
*qPI1*.*2*	ITA306xTOS14519	PI	1	150	146–154	K_id1022408	K_id1024503	11.6	7.3	-17.0	-8.9	-31.6
*qAfRGM1*.*4*	ITA306xTOS14519	PS	1	160	158–167	K_id1025292	K_id1027331	5.8	2.7	-1.8	-4.9	-8.5
*qPI2*.*1*	ITA306xTOG7106	PI	2	24	16–27	K_id2001831	K_id2001992	2.5	1.7	-4.3	16.1	-7.4
*qAfRGM2*	ITA306xBW348-1	PS	2	37	35–39	K_id2003730	id2004163	3.7	9.9	1.8	8.0	2.9
*qPI2*.*2*	ITA306xBW348-1	PI	2	45	44–46	id2004534	id2004650	2.8	9.3	0.4	4.6	0.7
*qAfRGM3*.*1*	ITA306xTOG7106	PS	3	10	8–12	K_9	K_id3001137	2.8	2.3	-14.5	-31.7	-28.3
*qPI3*	ITA306xTOG7106	PI	3	11	9–13	K_9	K_id3001137	3.9	2.2	-4.1	11.7	-6.2
*qAfRGM3*.*2*	ITA306xBW348-1	PS	3	73	64–78	K_id3007320	id3008199	2.8	7.7	-4.3	-5.0	-10.1
*qAfRGM4*	ITA306xTOS14519	PS	4	111	109–113	K_id4010825	K_id4011016	60.8	34.1	-11.7	4.0	-24.1
*qPI4*	ITA306xTOS14519	PI	4	111	109–115	K_id4010825	K_id4011016	25.6	14.9	-23.2	10.0	-48.5
*qAfRGM5*.*1*	ITA306xTOS14519	PS	5	24	9–34	K_id5001470	K_id5001534	3.0	1.3	1.7	-2.6	-7.7
*qAfRGM5*.*2*	ITA306xTOG7106	PS	5	46	43–52	K_id5003092	K_id5005055	2.5	2.3	-10.1	37.6	-16.2
*qPI5*	ITA306xTOG7106	PI	5	116	114–118	K_id5013749	K_id5014265	2.7	1.7	3.0	-12.7	-7.7
*qAfRGM5*.*3*	ITA306xTOG7106	PS	5	117	115–118	K_id5013749	K_id5014265	2.6	2.2	-11.0	38.8	-46.1
*qPI6*.*1*	ITA306xTOS14519	PI	6	37	35–43	K_id6003829	K_id6004862	15.4	8.8	18.2	10.0	34.2
*qAfRGM6*.*1*	ITA306xTOS14519	PS	6	59	57–61	K_id6006147	K_id6006336	4.0	1.9	1.6	4.0	0.7
*qAfRGM6*.*2*	ITA306xTOG7106	PS	6	82	80–84	K_id6011413	K_id6012080	2.7	2.2	-11.4	39.6	-17.2
*qPI6*.*2*	ITA306xTOG7106	PI	6	83	80–87	K_id6011413	K_id6012080	2.8	1.7	-4.7	9.3	-7.7
*qPI6*.*3*	ITA306xTOG7106	PI	6	107	104–110	K_id6014475	K_id6016093	3.2	2.2	-4.8	14.5	-7.3
*qAfRGM6*.*3*	ITA306xTOG7106	PS	6	112	109–115	K_id6016093	K_id6016484	2.7	2.0	-11.6	43.5	-46.1
*qAfRGM7*	ITA306xTOG7106	PS	7	83	81–86	K_id7003748	K_id7004343	2.7	2.4	-10.8	36.2	-18.2
*qPI7*	ITA306xTOG7106	PI	7	93	91–95	K_id7004343	K_id7004741	2.6	1.7	3.7	-13.4	-7.8
*qAfRGM11*	ITA306xTOG7106	PS	11	17	15–20	K_id11001422	K_id11001683	6.9	5.9	2.9	-55.1	12.2
*qPI11*	ITA306xTOG7106	PI	11	23	19–26	K_id11001422	K_id11001683	3.0	1.7	-0.3	4.9	-0.1

*PI (Pest Incidence): number of plants infested with galls; PS (Pest Severity); percentage of plants infested with galls.

**Difference = the difference between lines that were homozygous for the parent 2 (BW348-1, TOG7106 or TOS14519) alleles minus those lines homozygous for the ITA306 alleles at the two flanking markers of each QTL.

## Results

### Phenotypic evaluation and QTL for pest incidence

**Fig 2:**
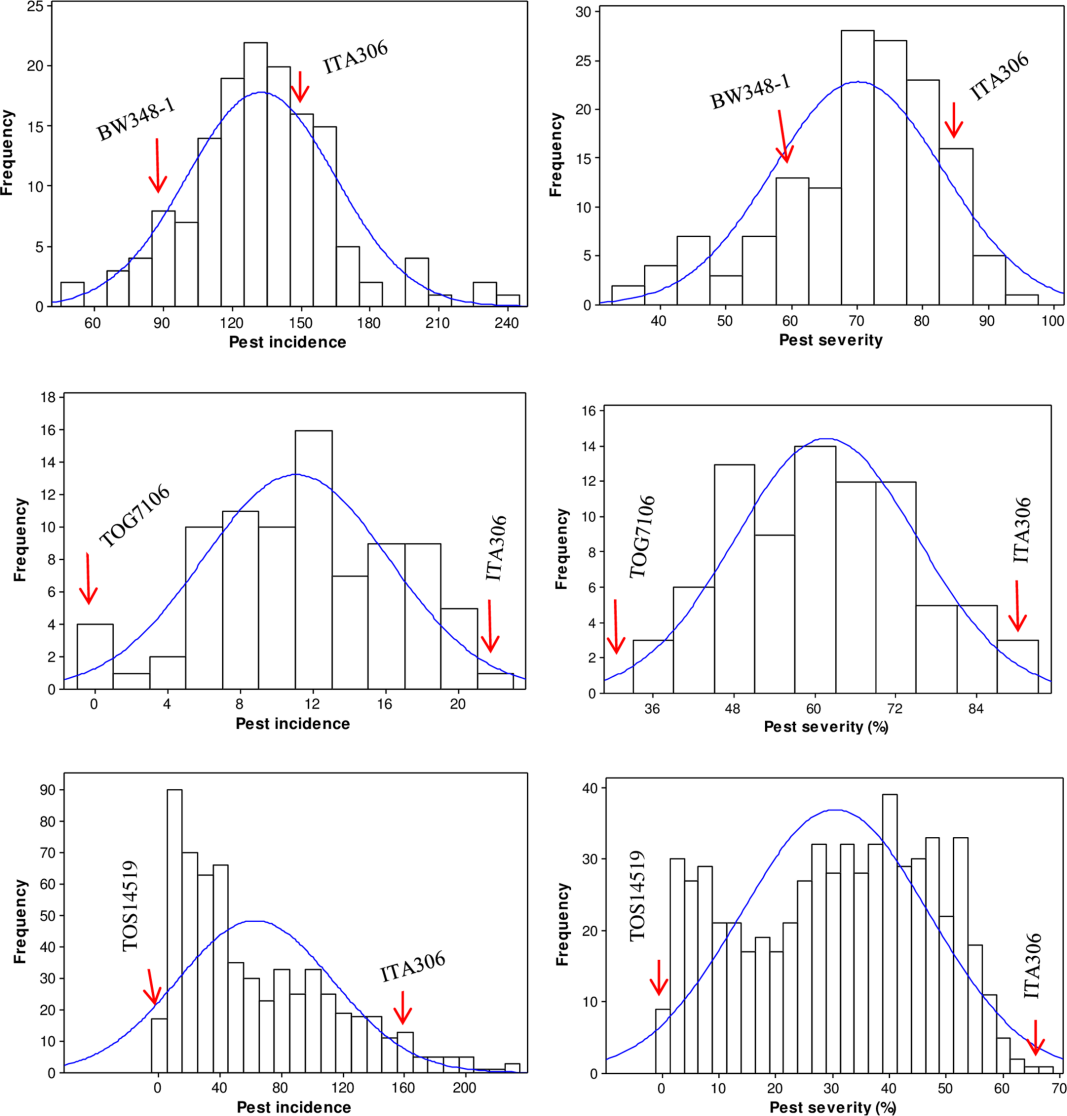
Frequency distribution of pest incidence (number of plants infested with galls) and pest severity (percentage of plants infested with galls) in the ITA306xBW348-1 (top), ITA306xTOG7106 (middle), and ITA306xTOS14519 (bottom) populations. Arrows indicate the values of two parents.

The mean phenotypic distributions for pest incidence and pest severity showed a continuous distributions in all three populations and were approximately normal (Fig 2) in both ITA306xTOG7106 and ITA306xBW348-1 populations. In the ITA306xTOS14519 population, however, the Shapiro-Wilk test rejected the hypothesis of normality (P < 0.010) for both pest incidence and severity, with the latter showing approximately bimodal distribution (Fig 2). Broad-sense heritability for pest incidence in the ITA306xTOG7106, ITA306xBW348-1 and ITA306xTOS14519 population was 0.44, 0.38 and 0.54, respectively. Heritability for pest severity was slightly higher than pest incidence: 0.49 for ITA306xTOG7106, 0.57 for ITA306xBW348-1 and 0.85 for ITA306xTOS14519.

**Fig 3:**
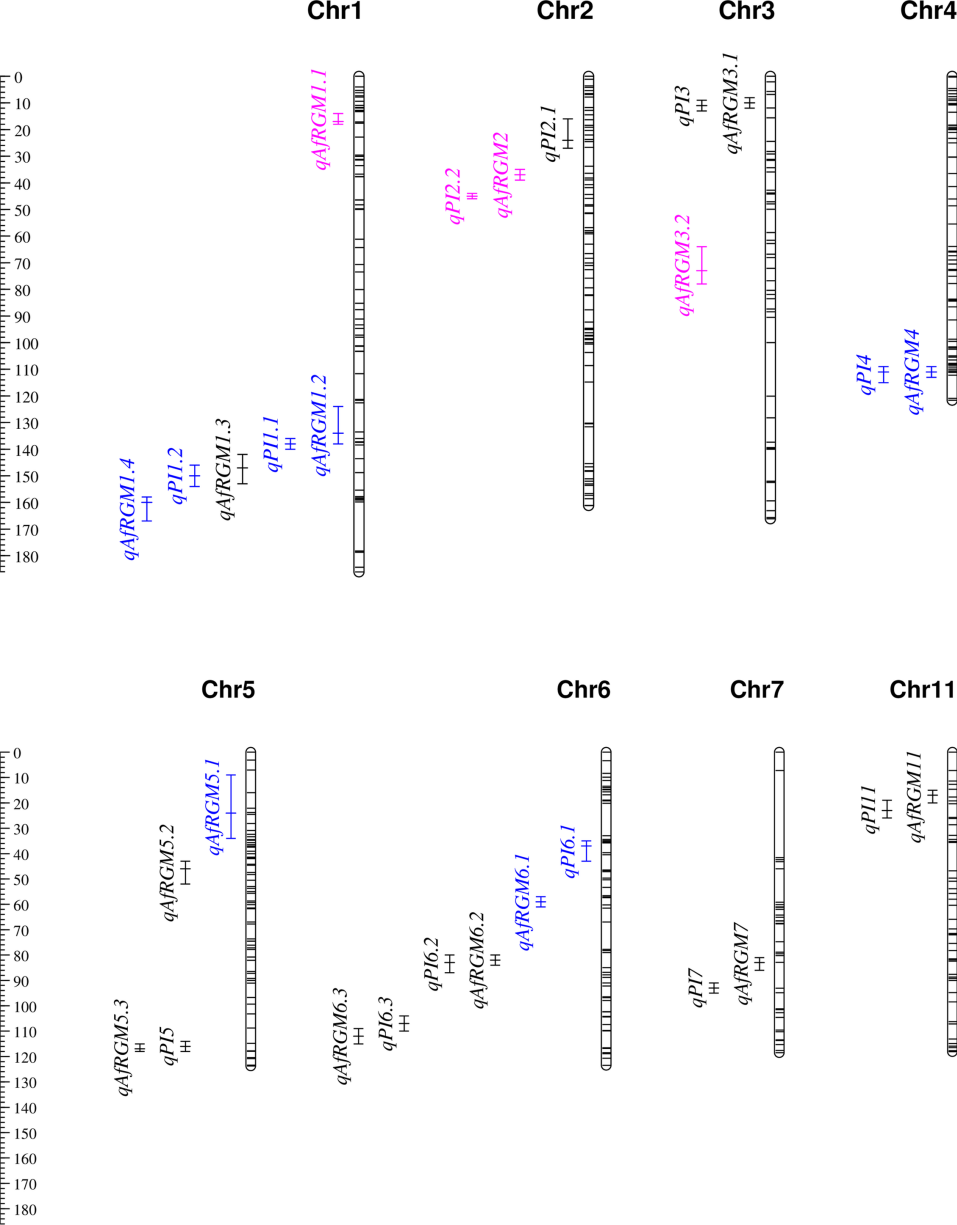
The positions of the 28 QTLs associated with African gall midge incidence (*qPI*) and severity (*qAfRGM*) in the ITA306xTOG7106 (black font), ITA306xBW348-1 (pink font) and ITA306xTOS14519 (blue font) populations. The vertical ruler on the left shows map position in centiMorgan. QTL are shown on the left side of each chromosome, with vertical bars indicating their 95% confidence interval. See Table 1 for details on each QTL and S1 Table for details on marker positions.

Pest incidence was one of the main traits used for measuring resistance to AfRGM across the three populations. From the three studies, CIM uncovered a total of 12 QTLs associated with pest incidence (Table 1), which includes one QTL for ITA306xBW348-1, four QTLs for ITA306xTOS14519 and seven QTLs for ITA306xTOG7106 population. All QTL for pest incidence exhibited mainly additive effects and QTL by QTL interactions were < 1.5% (data not shown). The QTL for pest incidence were distributed across chromosomes 1 to 7 and 11 (Fig 3), with the number of QTL per chromosome ranging from 1 to 3. No QTL for pest incidence was detected on chromosomes 8, 9, 10 or 12. The LOD score and percentage of phenotypic variance (R^2^) explained by each QTL for pest incidence varied from 2.5 to 25.6 and from 1.6 to 14.9%, respectively. Five of the 12 QTL associated with pest incidence had a LOD value > 3, of which two (*qPI3 and qPI6*.*3*) were in the ITA306xTOG7106 population and the other three *(qPI1*.*2*, *qPI4 and qPI6*.*1*) were identified in the ITA306xTOS14519 population. In the ITA306xTOG7106 population, the two QTL with LOD larger than 3 were mapped at 11 cM on chromosome 3 (*qPI3*) and at 107 cM on chromosome 6 (*qPI6*.*3*). Both *qPI3* and *qPI6*.*3* had a LOD score ranging from 3.2 to 3.9, individually explained 2.2% of the phenotypic variance for pest incidence, and originated from TOG7106. The BC_1_F_2_ families that were homozygous to the TOG7106 alleles at both flanking markers of each QTL showed between 6.2 and 7.3 fewer pest incidences than those families that were homozygous for the ITA306 alleles (Table 1).

The three QTL detected in the ITA306xTOS14519 population with LOD larger than 3 were mapped at 150 cM on chromosome 1 (*qPI1*.*2*), at 111 cM on chromosome 4 (*qPI4*) and at 37 cM on chromosome 6 (*qPI6*.*1*). The QTL on chromosome 1 (*qPI1*.*2*) had a LOD score of 11.6, explained 7.3% of the phenotypic variance for pest incidence, and originated from TOS14519. The F_2:3_ families that were homozygous to the TOS14519 alleles at both flanking markers (K_id1022408 and K_id1024503) for *qPI1*.*2* showed 31.6 fewer pest incidences than those families that were homozygous for the ITA306 alleles. The second QTL on chromosome 6 (*qPI6*.*1*) had a LOD score of 15.4, accounted for 8.8% of the phenotypic variance, and originated from ITA306. The F_2:3_ families that were homozygous to the ITA306 alleles at both flanking markers (K_id6004029 and K_id6004862) for *qPI6*.*1* showed on average 34.2 fewer pest incidences than those families that were homozygous for the TOS14519 alleles. The strongest QTL for pest incidence mapped on chromosome 4 (*qPI4*), had a LOD score of 25.6, explained 14.9% of the phenotypic variance, and originated from TOS14519. The F_2:3_ families that were homozygous to the TOS14519 alleles at both flanking markers (K_id4010825 and K_id4011016) for *qPI4* had on average 48.5 fewer pest incidences than those families that were homozygous for the ITA306 alleles (Table 1).

### QTL for AfRGM resistance

When QTL analyses were performed on each population independently using pest severity rather than pest incidence, CIM identified a total of sixteen QTL associated with pest resistance, which included three QTL in the ITA306xBW348-1 population, five QTL in the ITA306xTOS14519 population, and eight QTL in ITA306xTOG7106 population (Table 1). All QTL for pest resistance exhibited additive effects and QTL by QTL interactions were < 1% (data not shown). When results from the three populations were considered together, only eleven QTLs associated with pest resistance mapped either at the same position or within 4–22 cM confidence interval to those QTLs associated with pest incidence (Fig 3). The three QTLs associated with pest resistance in the ITA306xBW348-1 population mapped at 17 cM on chromosome 1 (*qAfRGM1*.*1*), at 37 cM on chromosome 2 (*qAfRGM2*) and at 73 cM on chromosome 3 (*qAfRGM3*.*2*); each QTL had a LOD score ranging from 2.8 to 3.7, individually explained between 6.8 to 9.9%, and altogether accounted for 24.4% of the phenotypic variance. The favorable alleles for both *qAfRGM1*.*1* and *qAfRGM2* originated from ITA306, while that of *qAfRGM3*.*2* from BW348-1. F_2:3_ families that were homozygous to the favorable alleles at the flanking markers of each QTL showed between 1.5 and 10.1% lower pest severity than those families that were homozygous to the unfavorable alleles (Table 1). One of the QTLs for pest resistance on chromosome 2 (*qAfRGM2*) mapped adjacent to the QTL for pest incidence (*qPI2*.*2*).

In the ITA306xTOS14519 population, the QTL for pest resistance on chromosome 4 (*qAfRGM4*) mapped at exactly the same position with the QTL for pest incidence (*qPI4*), three QTLs (*qAfRGM1*.*2*, *qAfRGM1*.*4* and *qAfRGM6*.*1*) mapped on the same chromosome with that of pest incidence (*qPI1*.*1*, *qPI1*.*2* and *qPI6*.*1*) but at slightly different positions that varied between 4 and 22 cM, while one QTL on chromosome 5 (*qAfRGM5*.*1*) was associated only with pest resistance (Table 1, Fig 3). However, the four QTLs that were associated with both pest incidence and pest resistance in the ITA306xTOS14519 population showed discrepancy both in LOD score and R^2^; both *qAfRGM1*.*2* and *qAfRGM4* showed 2–3 fold larger LOD and R^2^ values as compared with *qPI1*.*1* and *qPI4*, while *qAfRGM1*.*5* and *qAfRGM6*.*1* showed 2–5 fold reduction on LOD score and R^2^ as compared with *qPI1*.*2* and *qPI6*.*1* (Table 1). Each QTL associated with pest resistance in the ITA306xTOS14519 population had a LOD score and R^2^ that varied from 3.0 to 60.8 and from 1.3 to 34.1%, respectively, and altogether accounted for 44.3% of the phenotypic variance for pest severity. The major QTL for pest resistance (*qAfRGM4*) had a LOD score of 60.0 and explained 34.1% of the phenotypic variance. F_2:3_ families that were homozygous to the TOS14519 alleles at both flanking markers (K_id4010825 and K_id4011016) for *qAfRGM4* showed 24.1% lower pest severity than those families that were homozygous to the ITA306 alleles. The remaining four QTLs associated with pest resistance in the ITA306xTOS14519 population individually explained between 1.3 and 4.3% of the phenotypic variance, which is equivalent to a reduction on pest severity between 0.7 and 11.5%. The favorable alleles for *qAfRGM1*.*2* and *qAfRGM1*.*5* originated from TOS14519, while those for *qAfRGM5*.*1* and *qAfRGM6*.*1* originated from the ITA306.

In the ITA306xTOG7106 population, the eight QTLs associated with pest resistance included one each on chromosome 1 (*qAfRGM1*.*3*), chromosome 3 (*qAfRGM1*.*3*), chromosome 7 (*qAfRGM7*) and chromosome 11 (*qAfRGM11*), and two each on chromosome 5 (*qAfRGM5*.*2* and *qAfRGM5*.*3*) and chromosome 6 (*qAfRGM6*.*2* and *qAfRGM6*.*3*). All QTLs for pest resistance, except *qAfRGM1*.*3* and *qAfRGM5*.*2*, were in common (with the same flanking markers, and also similar LOD and R^2^ values) with those associated with pest incidence (Table 1). The LOD score and R^2^ values for the QTLs associated with pest resistance in the ITA306xTOG7106 population varied from 2.5 to 6.9 and from 2.0 to 5.9%, respectively, and altogether accounted for 22.1% of the phenotypic variance for pest resistance. The TOG7106 parent contributed the favorable alleles for all QTLs except *qAfRGM11*, which originated from ITA306. BC_1_F_2_ families that were homozygous to the favorable alleles at both flanking markers of each QTL showed between 12.2 and 46.1% lower pest severity than those families that were homozygous to the unfavorable alleles.

### Meta-analyses

All the 28 QTLs identified in individual populations (Table 1) were projected on the combined consensus map of 641 SNPs, with each chromosome harboring between two and six QTLs except chromosomes 8, 9, 10 and 12 (Fig 3). Both chromosomes 1 and 6 had the highest number of QTL clusters. The meta-analysis reduced the total number of QTLs from 28 to 17 mQTLs (Table 2), which is a 39.3% reduction compared with the individual studies. Excluding chromosomes 8, 9, 10 and 12 that did not harbor any QTL, the number of mQTLs identified on the other chromosomes varied from one on chromosomes 2, 4, 7 and 11 to four on both chromosomes 4 and 6 (Table 2). The mean phenotypic variance explained by each mQTL varied from 1.3 to 24.5%, while the 95% confidence intervals for the mQTLs varied between 0.2 and 25.0 cM, with an average of 5.5 cM. There was only one minor effect mQTL on chromosome 1 (MQTL1.3) that was common across two genetic backgrounds (ITA306xTOG7106 and ITA306xTOS14519), but none were associated in three genetic backgrounds. MQTL4.1 that mapped at 111 cM on chromosome 4 is the most important genomic region associated with AfRGM resistance, and explained 14.9 and 34.1% of the phenotypic variance for pest incidence and severity, respectively.

**Table 2:** Summary of the 17 meta QTLs associated with pest incidence and pest severity across three populations. See Table 1 for details on individual QTLs.

Chrom.	Meta QTL name	Meta QTL (K)	Predicted meta QTL position (cM)	Meta QTL 95% confidence interval (cM)	Initial number of QTLs	Mean initial confidence interval (cM)	Mean LOD score of the initial QTLs	Mean R^2^ for the initial QTLs	Change in the 95% confidence interval (cM)	Name of initial QTL
Chr1	MQTL1.1	1	17.0	4.0	1	4.0	3.0	6.8	0.0	*qAfRGM1*.*1*
Chr1	MQTL1.2	2	137.7	3.9	2	9.0	5.6	3.0	5.1	*qPI1*.*1*, *qAfRGM1*.*2*
Chr1	MQTL1.3	3	148.9	6.5	2	9.5	7.3	5.1	3.0	*qPI1*.*2*, *qAfRGM1*.*3*
Chr1	MQTL1.4	4	160.0	9.0	1	9.0	5.8	2.7	0.0	*qAfRGM1*.*4*
Chr2	MQTL2.1	1	42.9	0.2	3	5.7	3.0	7.3	5.5	*qPI2*.*1*, *qPI*2.2, qAfRGM2
Chr3	MQTL3.1	1	10.5	2.8	2	4.0	3.4	2.3	1.2	*qPI*3, qAfRGM3.1
Chr3	MQTL3.2	2	73.0	12.8	1	12.8	2.8	7.7	0.0	*qAfRGM3*.*2*
Chr4	MQTL4.1	1	111.0	0.7	2	5.0	43.2	24.5	4.3	*qPI*4, *qAfRGM4*
Chr5	MQTL5.1	1	24.0	25.0	1	25.0	3.0	1.3	0.0	*qAfRGM5*.*1*
Chr5	MQTL5.2	2	46.0	9.0	1	9.0	2.5	2.3	0.0	*qAfRGM5*.*2*
Chr5	MQTL5.3	3	116.6	0.4	2	3.5	2.7	1.5	3.1	*qPI5*, *qAfRGM5*.*3*
Chr6	MQTL6.1	1	37.0	8.0	1	8.0	15.4	8.8	0.0	*qPI6*.*1*
Chr6	MQTL6.2	2	59.0	4.0	1	4.0	4.0	1.9	0.0	qAfRGM6.1
Chr6	MQTL6.3	3	82.3	3.5	2	5.5	2.8	1.5	2.0	*qPI*6.2, *qAfRGM6*.*2*
Chr6	MQTL6.4	4	109.5	1.2	2	6.0	2.9	2.1	4.8	*qPI*6.3, *qAfRGM6*.*3*
Chr7	MQTL7.1	1	89.1	0.6	2	4.5	2.7	2.0	3.9	*qPI7*, *qAfRGM7*
Chr11	MQTL11.1	1	19.0	1.1	2	6.0	4.9	3.8	4.9	*qPI11*, *qAfRGM11*

## Discussion

*Oryza glaberrima* is found in diverse agro-ecosystems in Africa but, due to its poor agronomic performance [44], it has been largely replaced by high yielding *O*. *sativa* varieties. Although most of the improved *O*. *sativa* varieties grown in Africa have high yield potential, they are highly susceptible to AfRGM, which contributes to the increasing infestations of the pest in the region. Rice breeders have developed several AsRGM-resistant varieties using both conventional and marker-assisted breeding methods, but the majority of them are susceptible to AfRGM, while some others were not adopted due to other abiotic and biotic constraints in Africa. Many screening trials to identify sources of resistance to AfRGM have been undertaken in Africa, both under natural infestation in AfRGM hotspot areas and under artificial infestation in screen-houses. Results from such screening studies were useful in identifying some highly resistant and moderately resistant varieties, including TOG7106, TOS14519 and BW348-1. While TOG7106, an *O*. *glaberrima* variety, has often been rated as highly resistant or resistant to the AfRGM, there were contradictory reports regarding the AfRGM severity level for both TOS14519 and BW348-1, with some experiments rating them as highly resistant, but others rating them as moderately resistant or moderately susceptible [11, 17, 20]. There are several reasons for such discrepancies, including differences in the duration of infestation (some authors scored pest severity 40 to 45 days after infestation, while others scored 60 to 70 days after infestation), genotype-by-environment interaction, the screening facility used in the studies (natural infestation in field conditions versus artificial infestation in screen-houses), the ratio of plants to insects, climatic conditions, and differences in the biotypes of the gall midge used for phenotyping.

In order to uncover the genomic regions associated with AfRGM resistance in TOG7106, TOS14519 and BW348-1, we developed both interspecific (*O*. *sativa* x *O*. *glaberrima*) and intraspecific (*O*. *sativa* x *O*. *sativa*) mapping populations by crossing these three parents with different levels of resistance to AfRGM into the same highly AfRGM susceptible parent (ITA306) and evaluated the F_2:3_ and BC_1_F_2_ populations using the same phenotyping protocol and gall midge biotype(s). Composite interval mapping uncovered a total of 16 QTLs associated with AfRGM resistance, of which 8 QTLs were identified in the interspecific ITA306xTOG7106 population, with favorable alleles for all but one originating from the TOG7106 parent. The remaining QTLs were uncovered in the intraspecific ITA306xBW348-1 (3 QTLs) and ITA306xTOS14519 (5 QTLs) populations, with the TOS14519 parent contributing favorable alleles at four genomic regions. The proportion of phenotypic variance explained by each QTL associated with AfRGM in the ITA306xBW348-1, ITA306xTOG7106 and ITA306xTOS14519 populations varied from 6.8 to 9.9%, from 2.0 to 5.9% and from 1.3 to 34.1%, respectively. The most prominent QTL for AfRGM resistance, with the highest LOD score and *R*^2^ value (LOD = 60.0; *R*^2^ = 34.1%), was uncovered in the ITA306xTOS14519 population, which mapped at 111 cM on chromosome 4. Chromosome 4 has been found to be rich in plant defense-related genes [45]. Four major Asian gall midge (AsRGM) resistance genes (*Gm2*. *gm3*, *Gm6* and *Gm7*) have been mapped on chromosome 4 [14, 16, 45–48]. These results, together with ours, provide evidence that there may be a common genomic region on chromosome 4 for both African and Asian rice gall midge resistance, irrespective of the insect pest species and biotypes used for infestation.

In the ITA306xBW348-1 and ITA306xTOG7106 populations, both pest incidence and severity showed more quantitative and continuous frequency distribution with a single peak (Fig 2), implying that the traits are controlled by several genes or QTLs, each with small to moderate individual effect. The continuous distribution of pest incidence and severity in the ITA306xBW348-1 and ITA306xTOG7106 populations is concordant with the presence of three to eight genomic regions, each explaining between 2.0 and 9.9% of the phenotypic variance. In the ITA306xTOS14519 population, however, pest severity showed an approximately bimodal distribution (Fig 2) that may be predominantly controlled by a single gene, clusters of tightly linked genes, or few major effect QTLs [49, 50]. In the ITA306xTOS14519 population, we found four minor effect QTLs on chromosomes 1, 5 and 6 (*qAfRGM1*.*2*, *qAfRGM1*.*4*, *qAfRGM5*.*1* and *qAfRGM6*.*1*) that explained between 1.3 and 4.3% of the phenotypic variance and a single major effect QTL on chromosome 4 (*qAfRGM4*) that explained 34% of the phenotypic variance for pest severity (Table 1). In contrast to AsRGM, therefore, resistance to AfRGM seems to be regulated by a few major QTLs and several minor to moderate effect QTLs. The major QTL on chromosome 4 might consist of clusters of up to four genes (*Gm2*, *Gm3*, *Gm6* and *Gm7*) that regulate AsRGM resistance [14, 45–48]. Because of differences in computing map distances and the difficulty in aligning the positions of the SNP markers with that of AFLPs and SSRs markers, however, here we cannot accurately locate the exact position of this possible common genomic region.

Although it is possible to have a few common major genomic regions associated with resistance to both the Asian and African rice gall midge, as suggested on chromosome 4 above, the high vulnerability of the majority of the AsRGM-resistant varieties to AfRGM suggests that most of the genes that contribute to resistance to the AsRGM may be overcome by the AfRGM biotypes. For example, a total of 99 rice accessions that were rated as highly and moderately resistant to AsRGM were screened against AfRGM, but only two varieties showed moderate resistance to AfRGM, while all others were either moderately or highly susceptible [18]. Cultivation of varieties containing single resistance genes has frequently resulted in breakdown of resistance due to emergence of virulent biotypes of the insect. Pyramiding of two or more effective genes or QTLs in a single variety may lead to durable gall midge resistance. Nevertheless, *qAfRGM4* is a convincing candidate of further breeding efforts for AfRGM resistance.

The projection of QTLs from independent studies on a consensus map for meta-analysis allows us to ascertain whether the QTLs detected for different traits are the same, and if the QTLs are common across different mapping populations (genetic backgrounds). The meta-analysis reduced the number of QTLs associated with pest incidence and severity from 28 to 17, which was a reduction by 39% as compared with the individual studies. However, MQTL1.3 was the only common genomic region associated in two genetic backgrounds (TOG7106 and TOS14519), which explained on average 5.3% of the phenotypic variance (Table 2). All the other mQTLs were found to be background-specific, clearly suggesting the germplasm specificity of most genomic regions associated with AfRGM resistance.

Although *qAfRGM4* was detected only in the ITA306xTOS14519 population, it appears to be one of the strongest QTLs ever reported in the literature, with a LOD score of 60.0. Progenies that were homozygous to the TOS14519 alleles at both flanking markers (*K_id4010825* and *K_id4011016*) for *qAfRGM4* showed a reduction in AfRGM pest severity by 24.1% compared with those that were homozygous to the ITA306 alleles. We strongly believe that this QTL can be introgressed from the TOS14519 parents into other AfRGM susceptible but highly popular rice varieties widely grown in Africa using either F_2_ enrichment or marker-assisted backcrossing. We are currently fine-mapping and validating *qAfRGM4* using a large number of isogenic recombinants and high marker density.

## Conclusions

We identified a total of sixteen QTLs associated with AfRGM resistance, which included three QTL in the ITA306xBW348-1, five QTL in the ITA306xTOS14519 and eight QTL in ITA306xTOG7106 population. Each QTL had a LOD score ranging from 2.5 to 60.8 and explained between 1.3 and 34.1% of the phenotypic variance for pest resistance. The major effect genomic region for AfRGM resistance was identified in the ITA306xTOS14519 population, mapped at 111 cM on chromosome 4 (*qAfrGM4)*, had a LOD score of 60.0 and accounted for 34.1% of the total phenotypic variance. We are currently fine-mapping and validating *qAfrGM4* using a large number of isogenic recombinants. This is the first study that reports extensive results on AfRGM resistance, and will be extremely useful for other researchers working on AfRGM mapping for possible use in marker-assisted breeding and/or QTL cloning.

## Supporting Information

S1 Fig Schematic illustration of the different steps followed in developing the mapping populations. (PDF)

S1 Table Consensus linkage map positions for 641 SNP markers used for genotyping the three mapping positions. (PDF)

S2 Table Summary of model selection criteria in the meta-analysis. (PDF)
