# Supplementary figures and images for: QTL Mapping in Three Rice Populations Uncovers Major Genomic Regions Associated with African Rice Gall Midge Resistance
Source: PLoS One. 2016 Aug 10;11(8):e0160749. doi: 10.1371/journal.pone.0160749 (PMC4980037; doi:10.1371/journal.pone.0160749)

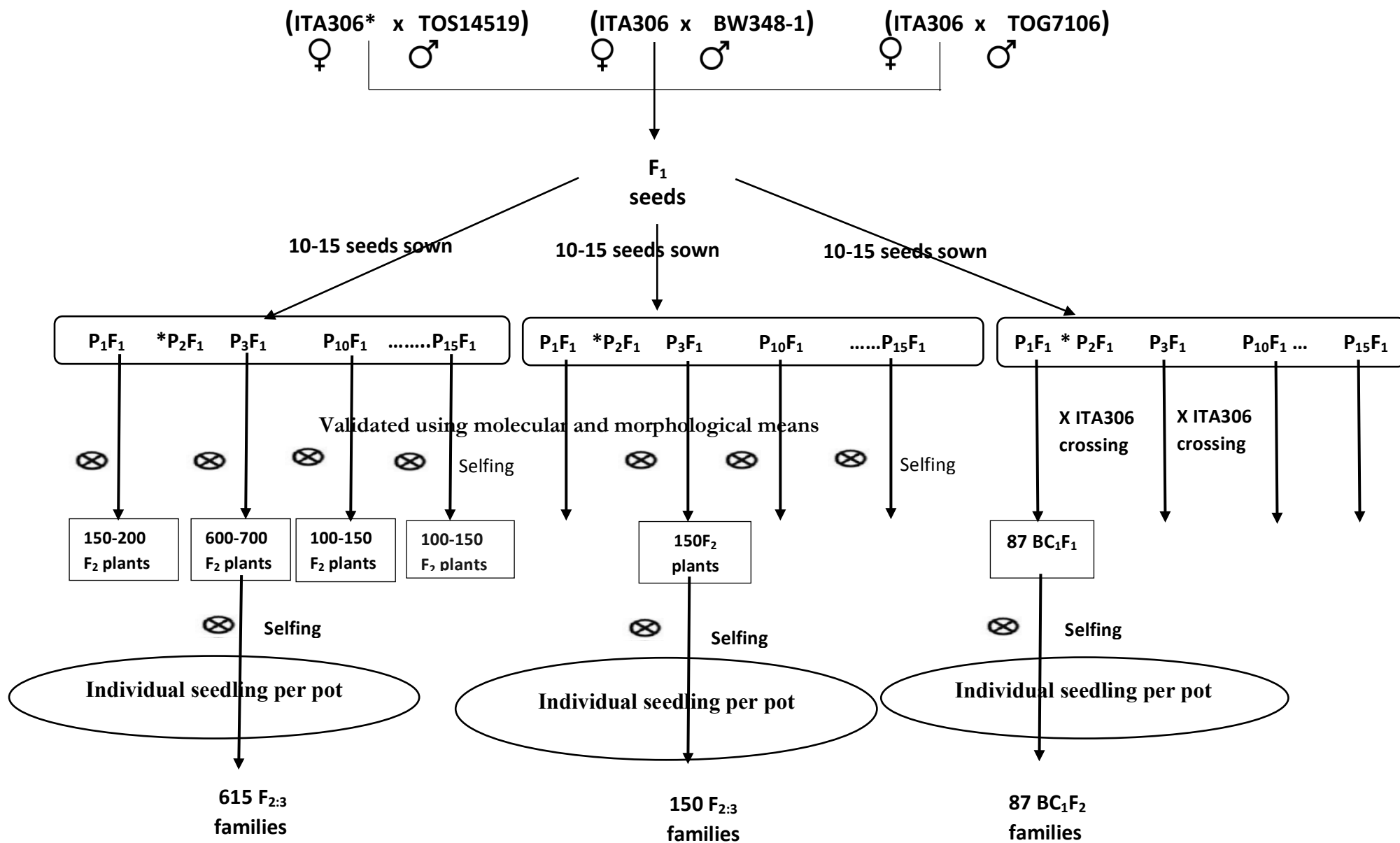

Supplement: S1 Fig — (PDF) [file pone.0160749.s001.pdf]
