# Supplementary material for: QTL Mapping in Three Rice Populations Uncovers Major Genomic Regions Associated with African Rice Gall Midge Resistance
Source: PLoS One. 2016 Aug 10;11(8):e0160749. doi: 10.1371/journal.pone.0160749 (PMC4980037; doi:10.1371/journal.pone.0160749)
Supplement: S1 Table — (PDF) [file pone.0160749.s002.pdf]

S1 Table. Consensus linkage map of 641 SNP markers used for genotyping three mapping rice populations.

S/N	chromosome	SNP	Cumulative position (cM)	Interval (cM)
1	1	id1000001	0.00	4.03
2	1	K_id1000223	4.03	1.22
3	1	id1000280	5.25	0.98
4	1	K_id1000556	6.23	1.09
5	1	K_id1000955	7.32	0.48
6	1	K_id1001073	7.80	1.55
7	1	id1001186	9.35	0.07
8	1	id1001235	9.42	1.51
9	1	K_id1001371	10.93	0.79
10	1	K_id1001516	11.72	0.16
11	1	id1001681	11.88	0.90
12	1	K_id1001973	12.78	0.25
13	1	K_id1002158	13.03	0.31
14	1	K_id1002308	13.34	3.80
15	1	id1002863	17.14	0.50
16	1	K_id1002899	17.64	5.20
17	1	K_id1003559	22.84	6.74
18	1	K_id1004420	29.58	0.51
19	1	id1004662	30.09	1.11
20	1	K_id1004849	31.20	0.15
21	1	K_id1004874	31.35	2.13
22	1	K_id1005125	33.48	3.19
23	1	id1005286	36.67	0.94
24	1	id1005463	37.61	0.08
25	1	K_id1005465	37.69	8.68
26	1	id1006571	46.37	0.13
27	1	K_id1006604	46.50	1.69
28	1	id1006691	48.19	1.57
29	1	id1006896	49.76	0.28
30	1	K_id1006954	50.04	11.17
31	1	K_id1007975	61.21	3.13
32	1	K_id1008267	64.34	6.40
33	1	K_id1008949	70.74	2.77
34	1	K_id1009867	73.51	6.62
35	1	K_id1011568	80.13	5.07
36	1	id1011905	85.20	2.43
37	1	id1012061	87.63	3.57
38	1	id1012539	91.20	2.13

39	1	K_id1012481	93.33	1.33
40	1	K_id1012666	94.66	2.50
41	1	K_id1013296	97.16	0.87
42	1	K_id1013342	98.03	3.19
43	1	K_id1013855	101.22	0.18
44	1	K_id1013967	101.40	1.78
45	1	K_id1014452	103.18	0.09
46	1	id1014783	103.27	8.42
47	1	id1015445	111.69	9.63
48	1	id1016524	121.32	0.32
49	1	id1016635	121.64	0.93
50	1	id1016882	122.57	10.90
51	1	K_id1019248	133.47	2.49
52	1	id1020204	135.96	1.27
53	1	K_id1020631	137.23	0.01
54	1	K_id1020667	137.24	0.21
55	1	K_id1020828	137.45	0.96
56	1	id1021344	138.41	5.14
57	1	K_id1022408	143.55	5.21
58	1	id1024167	148.76	6.63
59	1	K_id1024503	155.39	2.43
60	1	id1024936	157.82	0.45
61	1	K_id1024836	158.27	0.40
62	1	K_id1024973	158.67	0.39
63	1	K_id1025153	159.06	0.73
64	1	K_id1025292	159.79	18.36
65	1	K_id1027331	178.15	0.17
66	1	dd1002159	178.32	0.41
67	1	K_id1027554	178.73	5.57
68	1	K_id1028304	184.30	1.78
69	1	K_id1028615	186.08	
70	2	K_id2010169	0.00	0.04
71	2	id2000006	0.04	1.07
72	2	K_id2000096	1.11	2.52
73	2	id2000308	3.63	0.54
74	2	K_id2000405	4.17	1.22
75	2	K_id2000618	5.39	1.08
76	2	K_id2000835	6.47	0.37
77	2	id2000972	6.84	1.00
78	2	K_id2001102	7.84	3.81
79	2	id2001301	11.65	1.12
80	2	id2001432	12.77	1.76
81	2	K_id2001565	14.53	1.76
82	2	K_id2001831	16.29	2.11

83	2	K_id2001992	18.40	0.69
84	2	K_id2002110	19.09	1.17
85	2	K_id2002229	20.26	1.73
86	2	id2002319	21.99	1.72
87	2	K_id2002501	23.71	0.75
88	2	K_id2002589	24.46	2.20
89	2	K_id2002811	26.66	7.11
90	2	K_id2003393	33.77	2.62
91	2	K_id2003730	36.39	1.81
92	2	K_id2004100	38.20	0.72
93	2	id2004163	38.92	1.74
94	2	id2004323	40.66	0.01
95	2	id2004325	40.67	1.17
96	2	wd2000409	41.84	2.56
97	2	id2004534	44.40	1.36
98	2	id2004650	45.76	2.43
99	2	wd2000520	48.19	0.46
100	2	K_id2004711	48.65	0.00
101	2	K_id2004711	48.65	2.68
102	2	K_id2004970	51.33	0.21
103	2	id2005230	51.54	5.30
104	2	K_id2006486	56.84	1.18
105	2	K_id2006621	58.02	0.58
106	2	K_id2006795	58.60	0.56
107	2	id2007213	59.16	3.88
108	2	id2007264	63.04	3.52
109	2	id2007378	66.56	1.73
110	2	ud2001031	68.29	1.85
111	2	id2007446	70.14	0.96
112	2	K_id2007461	71.10	1.50
113	2	ud2001050	72.60	3.15
114	2	id2007494	75.75	3.63
115	2	id2007622	79.38	2.67
116	2	K_id2007951	82.05	0.26
117	2	id2008112	82.31	5.34
118	2	id2008705	87.65	4.60
119	2	id2009102	92.25	2.30
120	2	K_id2009463	94.55	0.25
121	2	id2009666	94.80	0.23
122	2	id2009758	95.03	0.04
123	2	id2009808	95.07	1.22
124	2	id2009964	96.29	1.00
125	2	id2010155	97.29	0.22
126	2	K_id2010169	97.51	1.00

127	2	K_id2010357	98.51	0.39
128	2	id2010481	98.90	1.12
129	2	K_id2010564	100.02	0.60
130	2	K_id2010624	100.62	3.07
131	2	K_id2010969	103.69	4.90
132	2	id2011535	108.59	6.23
133	2	id2012042	114.82	15.41
134	2	id2013434	130.23	0.22
135	2	id2013462	130.45	1.19
136	2	id2013634	131.64	13.71
137	2	id2014932	145.35	1.24
138	2	id2014987	146.59	1.53
139	2	ud2002021	148.12	0.15
140	2	K_id2015233	148.27	2.81
141	2	id2015384	151.08	0.88
142	2	id2015558	151.96	1.15
143	2	K_id2015767	153.11	0.51
144	2	id2015856	153.62	3.12
145	2	K_id2016060	156.74	0.59
146	2	id2016080	157.33	1.25
147	2	K_id2016156	158.58	2.51
148	2	id2016563	161.09	0.09
149	2	K_id2016584	161.18	0.00
150	2	K_id2016481	161.18	
151	3	id3000019	0.00	2.14
152	3	K_id3000111	2.14	3.60
153	3	id3000757	5.74	1.05
154	3	K_9	6.79	0.00
155	3	K_9	6.79	5.10
156	3	K_id3001137	11.89	3.73
157	3	id3001772	15.62	8.74
158	3	K_id3002805	24.36	3.79
159	3	id3003554	28.15	1.01
160	3	K_id3003294	29.16	2.02
161	3	id3003796	31.18	0.45
162	3	K_id3003697	31.63	2.64
163	3	K_id3003839	34.27	1.67
164	3	id3003914	35.94	6.89
165	3	id3004820	42.83	0.92
166	3	id3004859	43.75	0.49
167	3	id3004927	44.24	2.84
168	3	K_id3005145	47.08	0.84
169	3	K_id3005349	47.92	1.98
170	3	id3005879	49.90	8.80

171	3	K_id3006493	58.70	2.93
172	3	K_id3006721	61.63	1.17
173	3	K_id3006808	62.80	3.99
174	3	id3007315	66.79	1.27
175	3	K_id3007320	68.06	4.01
176	3	K_id3007703	72.07	4.74
177	3	K_id3007932	76.81	3.57
178	3	id3008199	80.38	1.48
179	3	id3008314	81.86	1.63
180	3	id3008419	83.49	3.95
181	3	K_id3009534	87.44	0.95
182	3	id3010378	88.39	0.01
183	3	K_id3010094	88.40	0.00
184	3	K_id3010094	88.40	2.05
185	3	K_id3010459	90.45	9.57
186	3	id3010993	100.02	20.03
187	3	K_id3011233	120.05	8.10
188	3	K_id3013480	128.15	9.26
189	3	id3014449	137.41	2.03
190	3	K_id3014586	139.44	0.28
191	3	K_id3014650	139.72	0.29
192	3	id3014942	140.01	11.94
193	3	id3016649	151.95	0.57
194	3	ud3001808	152.52	6.91
195	3	K_id3017469	159.43	3.62
196	3	id3018268	163.05	2.61
197	3	K_id3018439	165.66	0.50
198	3	K_id3018559	166.16	
199	4	K_id4000170	0.00	0.29
200	4	K_id4000179	0.29	4.31
201	4	id4000359	4.60	0.83
202	4	K_id4000429	5.43	1.18
203	4	K_id4000585	6.61	0.96
204	4	id4000869	7.57	0.89
205	4	id4001205	8.46	0.61
206	4	K_id4001365	9.07	1.09
207	4	id4001730	10.16	0.24
208	4	K_id4001733	10.40	0.35
209	4	K_id4001882	10.75	2.78
210	4	id4002166	13.53	4.77
211	4	K_id4002697	18.30	0.14
212	4	id4002844	18.44	0.70
213	4	id4002913	19.14	0.49
214	4	id4003259	19.63	0.27

215	4	id4003819	19.90	0.13
216	4	K_id4003727	20.03	1.10
217	4	K_id4003888	21.13	2.20
218	4	ud4001019	23.33	1.62
219	4	K_id4004294	24.95	5.32
220	4	id4004639	30.27	0.12
221	4	K_id4004644	30.39	0.01
222	4	K_id4004654	30.40	6.06
223	4	id4005072	36.46	4.98
224	4	id4005336	41.44	4.64
225	4	id4005474	46.08	2.52
226	4	id4005526	48.60	6.93
227	4	K_id4005867	55.53	8.34
228	4	K_id4007024	63.87	1.85
229	4	id4007172	65.72	0.26
230	4	K_id4007212	65.98	1.38
231	4	K_id4007444	67.36	1.60
232	4	id4007595	68.96	1.55
233	4	K_id4007764	70.51	1.95
234	4	id4007922	72.46	0.41
235	4	id4007959	72.87	2.21
236	4	K_id4008092	75.08	2.67
237	4	id4008430	77.75	5.93
238	4	K_id4008855	83.68	0.28
239	4	wd4003021	83.96	0.47
240	4	K_id4008998	84.43	2.14
241	4	K_id4009149	86.57	4.90
242	4	id4009390	91.47	7.27
243	4	K_id4009930	98.74	0.79
244	4	id4009997	99.53	2.11
245	4	K_id4010238	101.64	0.40
246	4	ud4002088	102.04	0.42
247	4	K_id4010396	102.46	2.57
248	4	K_id4010621	105.03	0.01
249	4	id4010651	105.04	0.42
250	4	K_id4010708	105.46	1.07
251	4	id4010800	106.53	1.29
252	4	id4010802	107.82	0.41
253	4	id4010815	108.23	0.10
254	4	K_id4010825	108.33	0.44
255	4	K_id4010876	108.77	0.76
256	4	id4010934	109.53	0.56
257	4	K_id4010985	110.09	0.64
258	4	K_id4011016	110.73	0.42

259	4	id4011112	111.15	1.06
260	4	K_id4011259	112.21	8.65
261	4	K_id4011696	120.86	0.72
262	4	K_id4011781	121.58	
263	5	K_id5001299	0.00	3.05
264	5	K_id5000128	3.05	4.07
265	5	K_id5000447	7.12	8.83
266	5	K_id5000953	15.95	6.13
267	5	K_id5001299	22.08	1.73
268	5	K_id5001470	23.81	0.68
269	5	K_id5001534	24.49	3.56
270	5	id5002131	28.05	2.83
271	5	id5002448	30.88	1.56
272	5	id5002496	32.44	0.96
273	5	id5002499	33.40	1.12
274	5	K_id5002468	34.52	0.22
275	5	id5002721	34.74	0.93
276	5	id5002861	35.67	0.82
277	5	K_id5002528	36.49	0.61
278	5	K_id5002650	37.10	0.35
279	5	K_id5002699	37.45	0.02
280	5	id5002971	37.47	1.63
281	5	id5003151	39.10	0.91
282	5	K_id5002960	40.01	1.43
283	5	K_id5003034	41.44	0.15
284	5	id5003312	41.59	0.23
285	5	K_id5003092	41.82	0.19
286	5	K_id5003134	42.01	2.22
287	5	wd5000601	44.23	0.36
288	5	id5003661	44.59	2.80
289	5	K_id5003638	47.39	0.79
290	5	K_id5003785	48.18	2.34
291	5	id5004346	50.52	2.46
292	5	K_id5004295	52.98	0.76
293	5	id5005953	53.74	1.28
294	5	K_id5005055	55.02	0.55
295	5	K_id5005495	55.57	3.08
296	5	K_id5006116	58.65	0.67
297	5	K_id5006236	59.32	0.79
298	5	K_id5006332	60.11	1.27
299	5	K_id5006470	61.38	0.29
300	5	ud5000744	61.67	5.32
301	5	id5007304	66.99	0.85
302	5	id5007323	67.84	5.77

303	5	wd5002636	73.61	0.89
304	5	K_id5007714	74.50	1.56
305	5	K_id5007981	76.06	1.12
306	5	K_id5008122	77.18	0.63
307	5	id5008574	77.81	0.01
308	5	id5008593	77.82	3.01
309	5	K_id5008590	80.83	1.28
310	5	K_id5008723	82.11	4.43
311	5	id5009280	86.54	0.67
312	5	K_id5009045	87.21	1.99
313	5	id5009481	89.20	0.84
314	5	id5009556	90.04	1.06
315	5	id5009700	91.10	5.60
316	5	id5010673	96.70	2.74
317	5	id5011044	99.44	3.76
318	5	K_id5011201	103.20	5.62
319	5	K_id5012489	108.82	5.99
320	5	K_id5013743	114.81	0.02
321	5	K_id5013749	114.83	2.92
322	5	K_id5014265	117.75	0.15
323	5	K_id5014338	117.90	2.52
324	5	id5014947	120.42	0.40
325	5	id5015048	120.82	2.56
326	5	K_id5014934	123.38	0.27
327	5	K_id5014986	123.65	
328	6	K_id6002100	0.00	0.00
329	6	K_id6002535	0.00	0.00
330	6	K_id6004038	0.00	3.44
331	6	K_id6000202	3.44	5.00
332	6	K_id6001206	8.44	1.46
333	6	K_id6001376	9.90	1.28
334	6	K_id6001535	11.18	2.24
335	6	K_id6002291	13.42	0.61
336	6	K_id6002535	14.03	0.61
337	6	id6002610	14.64	0.30
338	6	K_id6002687	14.94	0.80
339	6	K_id6002884	15.74	1.03
340	6	K_id6003050	16.77	2.17
341	6	K_id6003299	18.94	0.29
342	6	K_id6003341	19.23	0.95
343	6	K_id6003403	20.18	12.78
344	6	id6003627	32.96	1.48
345	6	K_id6003829	34.44	0.45
346	6	id6003932	34.89	0.38

347	6	K_id6004029	35.27	0.29
348	6	K_id6004038	35.56	0.28
349	6	id6004089	35.84	3.75
350	6	id6004385	39.59	0.85
351	6	K_id6004481	40.44	6.02
352	6	id6004849	46.46	0.21
353	6	K_id6004862	46.67	0.40
354	6	K_id6004868	47.07	2.66
355	6	K_id6004946	49.73	0.52
356	6	K_id6004969	50.25	3.07
357	6	K_id6005350	53.32	3.23
358	6	K_id6006089	56.55	0.51
359	6	id6006125	57.06	0.36
360	6	K_id6006147	57.42	2.74
361	6	K_id6006336	60.16	1.30
362	6	id6006537	61.46	5.52
363	6	K_id6009055	66.98	10.76
364	6	id6011280	77.74	0.41
365	6	K_id6011324	78.15	0.86
366	6	K_id6011413	79.01	5.97
367	6	K_id6012080	84.98	1.86
368	6	id6012273	86.84	0.99
369	6	id6012365	87.83	0.93
370	6	K_id6012487	88.76	2.20
371	6	id6012641	90.96	1.27
372	6	id6012967	92.23	4.29
373	6	K_id6013434	96.52	0.28
374	6	K_id6013529	96.80	1.14
375	6	K_id6013720	97.94	4.35
376	6	id6014523	102.29	0.10
377	6	K_id6014475	102.39	1.78
378	6	id6014779	104.17	0.19
379	6	id6014813	104.36	2.99
380	6	K_id6015421	107.35	2.39
381	6	id6016091	109.74	0.19
382	6	K_id6016093	109.93	6.67
383	6	K_id6016484	116.60	0.18
384	6	K_id6016490	116.78	1.73
385	6	K_id6016589	118.51	0.43
386	6	id6016613	118.94	1.77
387	6	K_id6016755	120.71	2.80
388	6	K_id6016918	123.51	
389	7	K_id7000063	0.00	7.20
390	7	K_id7000337	7.20	34.44

391	7	K_id7000798	41.64	0.73
392	7	K_id7000978	42.37	0.84
393	7	id7001153	43.21	2.77
394	7	id7001478	45.98	13.17
395	7	ud7001182	59.15	0.97
396	7	K_id7002758	60.12	0.83
397	7	K_id7002801	60.95	0.22
398	7	K_id7002848	61.17	0.81
399	7	K_id7002907	61.98	2.25
400	7	ud7001328	64.23	0.91
401	7	id7002946	65.14	1.40
402	7	id7003043	66.54	0.10
403	7	K_id7002978	66.64	0.89
404	7	K_id7003047	67.53	0.21
405	7	K_id7003059	67.74	7.02
406	7	id7003593	74.76	4.18
407	7	K_id7003748	78.94	0.56
408	7	id7004054	79.50	0.70
409	7	K_id7003994	80.20	2.68
410	7	K_id7004343	82.88	10.17
411	7	K_id7004741	93.05	1.81
412	7	K_id7004871	94.86	6.33
413	7	K_id7005306	101.19	0.33
414	7	id7005418	101.52	1.19
415	7	id7005449	102.71	1.84
416	7	K_id7005477	104.55	5.05
417	7	K_id7005611	109.60	0.58
418	7	id7005624	110.18	3.24
419	7	K_id7005655	113.42	0.24
420	7	id7005667	113.66	1.68
421	7	K_id7005828	115.34	2.27
422	7	K_id7006093	117.61	0.84
423	7	K_id7006185	118.45	
424	8	id8000118	0.00	4.04
425	8	id8000293	4.04	1.28
426	8	K_id8000315	5.32	2.63
427	8	id8000337	7.95	2.24
428	8	id8000536	10.19	1.45
429	8	id8000628	11.64	0.26
430	8	K_id8000666	11.90	0.88
431	8	id8000700	12.78	7.35
432	8	K_id8000876	20.13	0.45
433	8	id8000944	20.58	0.28
434	8	K_id8000975	20.86	8.28

435	8	K_id8001331	29.14	0.01
436	8	id8001345	29.15	1.26
437	8	wd8000422	30.41	1.04
438	8	K_id8001477	31.45	2.16
439	8	id8001604	33.61	2.01
440	8	K_id8001641	35.62	0.14
441	8	K_id8001667	35.76	5.21
442	8	K_id8002314	40.97	3.86
443	8	K_id8002632	44.83	1.72
444	8	K_id8002841	46.55	0.27
445	8	K_id8002954	46.82	0.69
446	8	K_id8003103	47.51	0.45
447	8	K_id8003220	47.96	1.62
448	8	K_id8003626	49.58	4.00
449	8	K_id8004400	53.58	1.74
450	8	K_id8004692	55.32	11.41
451	8	K_id8005445	66.73	1.46
452	8	K_id8005525	68.19	29.21
453	8	K_id8006885	97.40	0.03
454	8	K_id8006891	97.43	0.10
455	8	id8006881	97.53	0.85
456	8	ud8001656	98.38	0.74
457	8	K_id8006950	99.12	0.43
458	8	ud8001687	99.55	0.51
459	8	K_id8006997	100.06	0.98
460	8	K_id8007067	101.04	2.28
461	8	id8007144	103.32	0.72
462	8	K_id8007252	104.04	3.11
463	8	id8007295	107.15	5.27
464	8	id8007568	112.42	
465	9	id9000064	0.00	1.10
466	9	id9000783	1.10	0.43
467	9	K_id9001029	1.53	0.85
468	9	K_id9001308	2.38	5.69
469	9	id9001829	8.07	1.08
470	9	id9001883	9.15	3.88
471	9	id9002252	13.03	0.19
472	9	K_id9002255	13.22	1.31
473	9	id9002324	14.53	1.91
474	9	id9002357	16.44	1.86
475	9	id9002494	18.30	0.02
476	9	id9002497	18.32	0.21
477	9	K_id9002505	18.53	0.55
478	9	id9002529	19.08	0.21

479	9	K_id9002532	19.29	3.07
480	9	id9002547	22.36	1.24
481	9	id9002558	23.60	0.22
482	9	K_id9002563	23.82	6.56
483	9	id9002704	30.38	0.73
484	9	K_id9002721	31.11	1.08
485	9	id9002784	32.19	4.81
486	9	K_id9003188	37.00	1.85
487	9	id9003276	38.85	1.87
488	9	id9003470	40.72	0.19
489	9	K_id9003471	40.91	1.66
490	9	K_id9003562	42.57	3.72
491	9	K_id9003720	46.29	5.34
492	9	K_id9004100	51.63	0.10
493	9	id9004128	51.73	1.72
494	9	id9004297	53.45	4.11
495	9	K_id9004727	57.56	0.64
496	9	K_id9004788	58.20	2.97
497	9	id9005086	61.17	1.78
498	9	K_id9005502	62.95	1.48
499	9	id9005874	64.43	0.01
500	9	id9005890	64.44	4.53
501	9	K_id9006377	68.97	12.16
502	9	id9007203	81.13	1.72
503	9	K_id9007259	82.85	2.53
504	9	id9007315	85.38	3.83
505	9	K_id9007356	89.21	2.29
506	9	id9007525	91.50	0.25
507	9	K_id9007622	91.75	
508	10	K_id10000028	0.00	2.20
509	10	K_id10000350	2.20	1.32
510	10	K_id10000498	3.52	0.17
511	10	K_id10000561	3.69	1.77
512	10	K_id10000771	5.46	1.63
513	10	id10001118	7.09	2.14
514	10	K_id10001318	9.23	5.47
515	10	K_id10002180	14.70	0.23
516	10	K_id10002406	14.93	1.33
517	10	K_id10002660	16.26	1.20
518	10	K_id10002842	17.46	1.27
519	10	K_id10002912	18.73	1.82
520	10	ud10000715	20.55	0.44
521	10	id10002993	20.99	4.33
522	10	K_id10003620	25.32	1.34

523	10	id10003618	26.66	4.95
524	10	id10003870	31.61	4.13
525	10	K_id10004275	35.74	1.27
526	10	id10004327	37.01	1.03
527	10	id10004477	38.04	10.48
528	10	id10005328	48.52	2.39
529	10	id10005474	50.91	0.68
530	10	K_id10005716	51.59	1.11
531	10	id10005666	52.70	2.03
532	10	id10005979	54.73	0.10
533	10	K_id10006161	54.83	2.57
534	10	K_id10006328	57.40	0.60
535	10	K_id10006353	58.00	0.51
536	10	id10006323	58.51	15.87
537	10	K_id10006910	74.38	2.04
538	10	K_id10006963	76.42	5.90
539	10	K_id10007079	82.32	
540	11	K_id11011607	0.00	7.21
541	11	id11000414	7.21	4.14
542	11	id11000727	11.35	1.39
543	11	K_id11000784	12.74	1.83
544	11	id11000855	14.57	0.06
545	11	K_id11000858	14.63	3.06
546	11	K_id11000980	17.69	1.50
547	11	id11001134	19.19	1.09
548	11	K_id11001422	20.28	5.46
549	11	K_id11001683	25.74	0.39
550	11	K_id11001766	26.13	2.43
551	11	K_id11002205	28.56	4.28
552	11	ud11000299	32.84	1.58
553	11	id11002717	34.42	0.80
554	11	K_id11002764	35.22	0.35
555	11	K_id11002801	35.57	11.21
556	11	wd11000549	46.78	2.89
557	11	id11003480	49.67	1.24
558	11	id11003593	50.91	3.03
559	11	id11003686	53.94	1.80
560	11	K_id11004148	55.74	2.25
561	11	K_id11005447	57.99	2.38
562	11	K_id11005646	60.37	5.48
563	11	K_id11006022	65.85	3.86
564	11	K_id11006351	69.71	1.82
565	11	id11006550	71.53	0.25
566	11	id11006569	71.78	0.24

567	11	K_id11006588	72.02	3.36
568	11	id11006675	75.38	2.78
569	11	K_id11007012	78.16	3.32
570	11	K_id11007803	81.48	0.44
571	11	K_id11007840	81.92	0.46
572	11	id11007859	82.38	5.18
573	11	id11008193	87.56	0.06
574	11	K_id11008214	87.62	1.04
575	11	id11008358	88.66	0.54
576	11	K_id11008678	89.20	0.78
577	11	K_id11008862	89.98	4.79
578	11	K_id11009117	94.77	3.69
579	11	K_id11009358	98.46	7.86
580	11	K_id11010245	106.32	0.89
581	11	K_id11010309	107.21	5.85
582	11	id11010544	113.06	1.83
583	11	id11010893	114.89	1.05
584	11	K_id11011159	115.94	0.58
585	11	K_id11011285	116.52	0.02
586	11	id11011243	116.54	1.17
587	11	K_id11011505	117.71	0.30
588	11	id11011578	118.01	0.07
589	11	K_id11011607	118.08	
590	12	id12000292	0.00	6.33
591	12	K_id12000266	6.33	0.92
592	12	K_id12000592	7.25	9.48
593	12	ud12000118	16.73	0.58
594	12	id12001344	17.31	2.94
595	12	id12001628	20.25	2.97
596	12	K_id12001224	23.22	0.44
597	12	K_id12001321	23.66	2.37
598	12	id12002057	26.03	5.52
599	12	K_id12001996	31.55	7.11
600	12	id12003141	38.66	0.13
601	12	id12003239	38.79	0.99
602	12	K_id12002778	39.78	0.12
603	12	ud12000534	39.90	0.24
604	12	id12003789	40.14	3.42
605	12	id12004974	43.56	1.27
606	12	wd12002512	44.83	4.80
607	12	K_id12004885	49.63	1.96
608	12	K_id12005302	51.59	1.16
609	12	K_id12005428	52.75	3.50
610	12	K_id12005677	56.25	2.33

611	12	K_id12005892	58.58	0.38
612	12	K_id12005991	58.96	1.67
613	12	K_id12006216	60.63	1.83
614	12	id12006792	62.46	0.97
615	12	K_id12006515	63.43	0.14
616	12	K_id12006560	63.57	3.13
617	12	id12007341	66.70	2.14
618	12	K_id12006801	68.84	1.31
619	12	K_id12007081	70.15	0.30
620	12	K_id12007161	70.45	1.23
621	12	K_id12007231	71.68	0.46
622	12	id12007672	72.14	1.57
623	12	K_id12007407	73.71	0.46
624	12	K_id12007506	74.17	1.88
625	12	K_id12007577	76.05	5.09
626	12	id12008134	81.14	0.27
627	12	id12008145	81.41	2.02
628	12	id12008347	83.43	1.34
629	12	id12008557	84.77	0.35
630	12	K_id12007988	85.12	1.32
631	12	id12008675	86.44	0.22
632	12	id12008700	86.66	2.47
633	12	K_id12008285	89.13	0.56
634	12	K_id12008328	89.69	1.88
635	12	id12009192	91.57	0.73
636	12	K_id12008641	92.30	0.44
637	12	K_id12008665	92.74	2.90
638	12	ud12001525	95.64	0.97
639	12	id12009787	96.61	7.58
640	12	K_id12009820	104.19	2.48
641	12	K_id12010152	106.67	
