# Supplementary material for: QTL Mapping in Three Rice Populations Uncovers Major Genomic Regions Associated with African Rice Gall Midge Resistance
Source: PLoS One. 2016 Aug 10;11(8):e0160749. doi: 10.1371/journal.pone.0160749 (PMC4980037; doi:10.1371/journal.pone.0160749)
Supplement: S2 Table — (PDF) [file pone.0160749.s003.pdf]

S2 Table. Summary of model selection criteria in the meta-analyses. For each model (AIC, AICs, AIC3, BIC and AWE) within a chromosome, the value that best predicted the number of meta QTLs (K) is shown in bold face. For each chromosome, the best K is the one that had the lowest values at least in three models.

**Chrom**	**K**	**AIC**	**AICc**	**AIC3**	**BIC**	**AWE**
Chr1	1	1964.1	1965.1	1965.1	1963.9	1968.7
Chr1	2	123.7	135.7	126.7	123.1	135.7
Chr1	3	52.6	52.6	57.6	51.5	67.2
Chr1	**4**	**45.2**	**45.2**	**52.2**	**43.7**	65.0
Chr1	5	54.7	54.7	63.7	52.8	80.0
Chr1	6	50.0	50.0	56.0	48.7	**58.3**
Chr2	**1**	**18.9**	**18.9**	**21.9**	**16.2**	**21.9**
Chr2	2	35.1	35.1	38.1	32.3	40.8
Chr2	3	104.0	108.0	105.0	103.1	107.2
Chr3	1	304.9	308.9	305.9	304.0	308.1
Chr3	**2**	**18.4**	**18.4**	**21.4**	**15.7**	**23.7**
Chr3	3	18.8	18.8	21.8	16.1	24.2
Chr4	**1**	**6.6**	**6.6**	**7.6**	**5.3**	**9.0**
Chr4	2	8.6	8.6	10.6	6.0	13.3
Chr5	1	1087.2	1089.2	1088.2	1086.6	1091.0
Chr5	2	34.9	34.9	37.9	33.1	40.7
Chr5	**3**	**29.6**	**29.6**	**33.6**	**27.2**	**35.3**
Chr5	4	31.2	31.2	36.2	28.1	41.7
Chr6	1	1638.0	1639.0	1639.0	1637.8	1642.6
Chr6	2	507.3	519.3	510.3	506.6	513.4
Chr6	3	137.1	137.1	142.1	136.1	146.9
Chr6	**4**	**50.9**	**50.9**	**57.9**	**49.5**	**67.1**
Chr6	5	52.3	52.3	61.3	50.5	75.0
Chr6	6	46.7	46.7	52.7	45.4	54.9
Chr7	**1**	**11.0**	**11.0**	**13.0**	**8.4**	**13.0**
Chr7	2	43.7	43.7	44.7	42.4	46.1
Chr11	**1**	**12.1**	**12.1**	**14.1**	**9.5**	**14.1**
Chr11	2	14.8	14.8	15.8	13.5	17.2
